# Physical exercise and bedtime procrastination among college students: mediating roles of self-control and mobile phone addiction

**DOI:** 10.3389/fpsyg.2025.1630326

**Published:** 2025-07-17

**Authors:** Yangping Wang, Xudong Wang, Shanshan Wu, Weijie Zhang, Jingjing Li, Bing Liu

**Affiliations:** ^1^Physical Education College, Shanghai University, Shanghai, China; ^2^Sports Science Research Center, Shanghai University, Shanghai, China; ^3^The High School Affiliated to East China University of Political Science and Law, Shanghai, China

**Keywords:** bedtime procrastination, self-control, mobile phone addiction, physical exercise, college students

## Abstract

**Background:**

Sleep is a crucial foundation for maintaining both physical and mental health. Bedtime procrastination has been identified as a significant behavioral factor contributing to decreased sleep quality. Research has identified bedtime procrastination as a prevalent issue among college students, posing significant challenges to their overall health. Regular physical exercise, as a positive lifestyle intervention, may mitigate procrastination behaviors. However, the specific effects of physical exercise on bedtime procrastination among college students, as well as the underlying mechanisms, remain unclear and warrant further investigation.

**Objective:**

This study aims to examine the effects of physical exercise on bedtime procrastination in college students and to analyze the mediating roles of self-control and mobile phone addiction.

**Methods:**

A total of 1,000 college students from four universities were surveyed using the Physical Activity Rating Scale (PARS-3), the Bedtime Procrastination Scale (BPS), the College Student Self-Control Scale (CSSCS), and the Mobile Phone Addiction Index (MPAI) through online questionnaires.

**Results:**

(1) Physical exercise was positively correlated with self-control (*p* < 0.001) and negatively correlated with mobile phone addiction (*p* < 0.001). Moreover, a significant negative association was identified between self-control and both mobile phone addiction and bedtime procrastination among college students (*p* < 0.001). Furthermore, mobile phone addiction was positively correlated with bedtime procrastination (*p* < 0.001). (2) The total effect of physical exercise on bedtime procrastination was significant (*β* = −0.137, 95% CI = [−0.191, −0.083]); moreover, physical exercise alleviated bedtime procrastination through a chain mediation effect involving self-control and mobile phone addiction (*β* = −0.019, 95% CI = [−0.032, −0.010]), indicating that physical exercise mitigated mobile phone addiction by fostering self-control, thereby alleviating bedtime procrastination.

**Conclusion:**

Physical exercise significantly reduces bedtime procrastination among college students. Self-control and mobile phone addiction serve as mediators in the relationship between physical exercise and bedtime procrastination.

## 1 Introduction

As a core element of maintaining both physical and mental health, sleep quality significantly impacts an individual’s psychological state and overall quality of life. The university stage marks a critical period in adolescents’ transition to adulthood, during which sufficient and high-quality sleep serves as a fundamental basis for maintaining daily functioning and supporting academic performance. Bedtime procrastination refers to the behavior of individuals who intentionally delay their predetermined bedtime in the absence of external environmental or physiological factors. This phenomenon is a significant contributor to the decline in sleep quality among college students. Bedtime procrastination encompasses both “bedtime delay” and “sleep time delay,” while also reflecting the influence of modern lifestyle factors, such as the widespread use of the Internet and electronic devices, on individuals’ sleep behaviors (Kroese et al., 2014; Nauts et al., 2016; Xu, 2017). Research indicates that bedtime procrastination is prevalent among modern students, with over half of respondents exhibiting irrational late sleeping behaviors (Chinese Sleep Research Society, 2022).

Bedtime procrastination can adversely affect an individual’s physical and mental health. On the one hand, persistent bedtime procrastination tends to cause disturbances in sleep duration and rhythm, which in turn induces decreased attention, memory loss, and cognitive dysfunction (Cox and Olatunji, 2016; Irwin, 2015). On the other hand, bedtime procrastination is also closely related to emotional problems and is an important risk factor for psychological symptoms such as anxiety and depression (Cox and Olatunji, 2016; Sirois and Pychyl, 2013). In addition, bedtime procrastination not only affects academic performance and daily living efficiency of college students, but may also increase the risk of chronic diseases such as metabolic syndrome (Kamphorst et al., 2018). Therefore, investigating bedtime procrastination among college students holds substantial practical significance, as it may inform the development of effective intervention strategies to reduce procrastinatory sleep behaviors and ultimately enhance their overall quality of life.

## 2 Literature review

### 2.1 Physical exercise and bedtime procrastination

Current studies on bedtime procrastination in college students mainly focus on the behavioral characteristics and prevalence, psychological mechanisms and influencing factors, intervention paths and protective factors of bedtime procrastination. Intervention strategies for addressing bedtime procrastination among college students primarily include behavioral regulation, cognitive-behavioral interventions, emotional regulation and mindfulness-based approaches, as well as lifestyle modifications. Among these, physical exercise, cognized as a positive behavioral intervention, has been shown to enhance sleep quality at both physiological and psychological levels. Empirical studies suggest that engaging in regular physical activity can modify daily routines and significantly reduce the tendency to bedtime procrastination among college students (Cheng et al., 2025). Thirty minutes of moderate-intensity exercise during the day can increase the duration of slow-wave sleep by 22% and reduce the incidence of bedtime procrastination by 18.6% (Zhu and Wu, 2023). The empirical study of college students also pointed out the positive effect of physical exercise on bedtime procrastination. Some studies have pointed out that aerobic exercise can effectively reduce the tendency of students to delay falling asleep (Wang and Du, 2024), and other studies have pointed out that moderate intensity of regular exercise can indirectly promote the management of bedtime behaviors by improving heart rate variability and emotional regulation ability (Tseng et al., 2020). Empirical studies have demonstrated that physical exercise is negatively associated with both anxiety and bedtime procrastination. In addition, physical exercise and anxiety jointly serve as a chain mediating mechanism in the association between mobile phone addiction and bedtime procrastination (Meng et al., 2024). Therefore, hypothesis 1 is put forward: physical exercise negatively affects the level of bedtime procrastination among college students.

### 2.2 Mediating role of self-control between physical exercise and bedtime procrastination

Bedtime procrastination is a behavior involving multiple mechanisms such as dysrhythmia, digital media dependence and executive dysfunction, and is essentially a typical manifestation of self-control resource depletion (You et al., 2020; Zhou and Huang, 2024). Good self-control helps to improve sleep quality and sleep duration (Exelmans and Van den Bulck, 2018), while improving individual self-control helps individuals develop good sleep habits (Guarana et al., 2021). Current research confirms that physical exercise can have an impact on bedtime procrastination behavior. For example, physical exercise can significantly improve an individual’s emotional regulation ability by activating the cognitive control function of the prefrontal cortex (Giles et al., 2018; Wang et al., 2024), and enhance an individual’s execution of the sleep plan (Zhong and Chu, 2013). Therefore, hypothesis 2 is proposed: self-control plays a mediating role in the influence of physical exercise on college students’ bedtime procrastination.

### 2.3 Mediating role of mobile phone addiction between physical exercise and bedtime procrastination

Physical exercise plays a potential role in regulating mobile phone addiction behavior. Research indicates that the higher the frequency of physical exercise, the lower the level of mobile phone addiction, and there is a significant negative correlation between the two, that is, the university students who regularly participate in physical exercise have a significantly lower mobile phone use time and addiction tendency than the non-exercise group (Li et al., 2023; Yang et al., 2021; Zeng et al., 2022). The reasons are mainly attributed to the following aspects: (1) Physical exercise can effectively occupy the free time of individuals, replace part of the mobile phone use time, so as to reduce their dependence on mobile phones; (2) The positive emotional experience and social interaction during exercise can help meet the psychological needs of individuals and reduce their tendency to seek compensatory satisfaction through mobile phones (Guo et al., 2022; Zeng et al., 2022; Zhou, 2015). In addition, the study found that mobile phone addiction has a significant positive predictive effect on college students’ bedtime procrastination, that is, the stronger the dependence on mobile phones, the easier it is to delay the time of falling asleep, thus affecting the rest and rest at night (Wei, 2023). At the same time, relevant studies have pointed out that physical exercise can relieve stress, improve emotional state and enhance self-control ability, reduce dependence on mobile phones, reduce the frequency of mobile phone use at night, and reduce bedtime procrastination caused by excessive use of mobile phones (Yang et al., 2020). Based on this, hypothesis 3 is proposed in this study: mobile phone addiction plays a mediating role in the influence of physical exercise on college students’ bedtime procrastination.

### 2.4 The chain mediating role of self-control and mobile phone addiction on the relationship between physical exercise and bedtime procrastination

Empirical studies have shown that the pathway of mobile phone addiction between physical exercise and bedtime procrastination is not always significant. In some multi-mediating models, the mediating effect of mobile phone addiction is not significant, which may be influenced by the interaction of multiple variables such as emotional state and self-control ability. It has also been pointed out that the effect of physical exercise on bedtime procrastination is more dependent on internal regulatory mechanisms such as self-control than on reducing mobile phone addiction alone (Werner and Ford, 2023). Studies have shown that people with weak self-control are prone to addiction to mobile terminal use (Nie et al., 2013), forming a behavior closed loop of ‘out-of-control-addiction-staying up late’, which is highly related to the common phenomenon of 97.24% college students using mobile phones before going to bed (Ye et al., 2021). Therefore, this study proposed hypothesis 4: self-control and mobile phone addiction play a chain mediating role in physical exercise and bedtime procrastination of college students.

**Figure 1:**
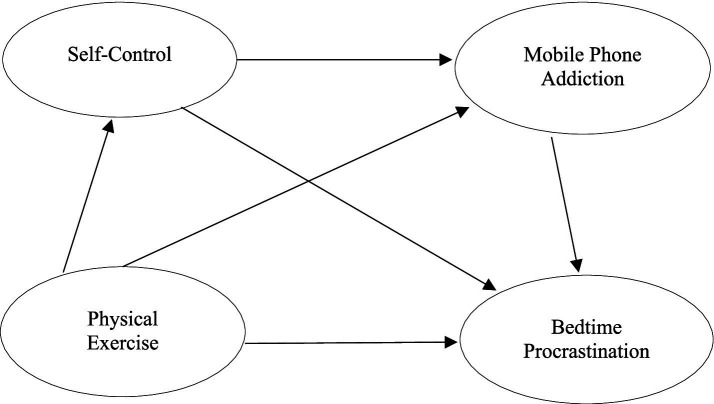
Hypothetical model of self-control and mobile phone addiction in the relationship between physical exercise and bedtime procrastination.

Based on this, the present study will explore the effect of physical exercise on bedtime procrastination of college students, and further reveal the role of self-control and mobile phone addiction in the influence of physical exercise on bedtime procrastination, in order to provide a theoretical basis for intervention strategies for bedtime procrastination, and effectively delay or reduce the physical and mental health problems of college students induced by bedtime procrastination (Figure 1).

## 3 Methods

### 3.1 Participants and procedures

Following approval by the Ethics Committee of Shanghai University (ECSHU:2025–019), students from four universities located in Shanghai, Tianjin, Zhejiang Province, and Anhui Province were selected as research participants using a convenience sampling method. With the help and organization of physical education teachers in each school, the students were selected as the unit before physical education class. Students were informed of the purpose of the survey, privacy protection, data storage and other information, and electronic questionnaires were issued using the questionnaire star platform. After students filled in the informed consent form online, they completed the questionnaires anonymously under the guidance of teachers. A total of 1,000 questionnaires were collected. Questionnaires were excluded if the completion time was less than 4 min (as determined by the average time in the pre-survey) or if five or more consecutive items had identical responses. After data cleaning, 935 valid responses were retained for analysis, including 435 males (46.5%) and 500 females (53.5%).

### 3.2 Research tools

#### 3.2.1 Physical activity rating scale-3

Physical activity rating scale-3 (PARS-3) was used to measure the physical exercise level of college students (Liang, 1994). This scale is designed to investigate the three dimensions of individual physical exercise intensity (I), single exercise time (T) and exercise frequency (F), with one question in each dimension and a score ranging from 1 to 5. According to the criteria of the scale, the physical exercise score = I* (T-1) *F. The physical activity score ranges from 0 to 100, with low level of physical exercise ≤19 points, medium level of physical exercise with 20–42 points, and high level of physical exercise ≥43 points.

#### 3.2.2 Bedtime procrastination scale

Bedtime procrastination scale (BPS) compiled by Kroese et al. (2014), and revised by Ma et al. (2021), is used to measure bedtime procrastination of college students. The scale consists of nine items, such as “I went to bed later than I expected” and “I tried to go to bed on time, but I just could not.” The scale uses a 5-point Likert scale, where 1 means “never” and 5 means “always.” The average score of all the questions is the score of bedtime procrastination, and the higher the score, the more serious the bedtime procrastination behavior. The Cronbach’s α coefficient of this scale in this study was 0.876.

#### 3.2.3 Self-control scale

The Self-Control Scale (SCS), originally developed by Tangney et al. (2004) and later revised by Tan and Guo (2008), was used to assess the self-control ability of college students. The scale consists of 19 items, including 4 positive items (e.g., “I am good at resisting temptation”) and 15 negative items (e.g., “I have trouble concentrating”). Each item is rated on a 5-point Likert scale, where 1 means “never” and 5 means “always,” yielding a total score between 19 and 95. After reverse scoring the negative items, higher total scores indicate greater self-control. In the present study, the Cronbach’s α coefficient for the scale was 0.891.

#### 3.2.4 Mobile phone addiction index

Mobile phone addiction index (MPAI) compiled by Leung (2008) was used to measure college students’ mobile phone addiction (Leung, 2008), which includes 17 items, such as “You will feel uneasy without your mobile phone,” “You will become anxious if you have not checked mobile phone messages or turned on your mobile phone for a period of time.” The scale uses a 5-point Likert scale, where 1 means “never” and 5 means “always.” The average score of all the questions is calculated, and the higher the score, the more severe of mobile phone addiction. The Cronbach’s α coefficient of this scale in this study was 0.902.

### 3.3 Data analysis

Data analysis was conducted using SPSS 26.0 software. First, a test for common method bias, descriptive statistics and Pearson’s correlation analysis were performed. Additionally, Model 6 of the SPSS Process macro (4.1 version) was utilized to examine the mediating effects among the variables using the Bootstrap method, in order to validate the proposed hypothetical model.

## 4 Results

### 4.1 Common method bias test

Harman’s single factor test was conducted to assess common method bias. The results revealed 11 factors with eigenvalues greater than 1, and the largest variance explained by a single factor was 24.23%, which is below the critical threshold of 40%. Therefore, there was no serious common method bias in this study.

### 4.2 The descriptive statistics and correlation analysis

The average score of each variable was used to conduct a Pearson’s correlation analysis, and the results showed that physical exercise was positively correlated with self-control, and negatively correlated with mobile phone addiction and bedtime procrastination. There was a significant negative correlation between self-control and mobile phone addiction. Moreover, mobile phone addiction was positively correlated with bedtime procrastination (Table 1).

**Table 1:** Descriptive statistics and correlation analysis.

Variables	M	SD	Physical exercise	Bedtime procrastination	Self-control	Mobile phone addiction
Physical exercise	19.26	21.73	1			
Bedtime procrastination	3.25	0.80	−0.11**	1		
Self-control	3.10	0.58	0.17**	−0.53**	1	
Mobile phone addiction	2.75	0.70	−0.11**	0.45**	−0.62**	1

M, Mean; SD, Standard Deviation. **p < 0.01.

### 4.3 Analysis of the mediating role of physical exercise on mobile phone addiction

Using the SPSS Process macro (version 4.1), this study examined the validity of a chain mediation model. In this model, bedtime procrastination served as the dependent variable, physical exercise as the independent variable, and self-control along with mobile phone addiction as mediating variables (Figure 1).

**Figure 2:**
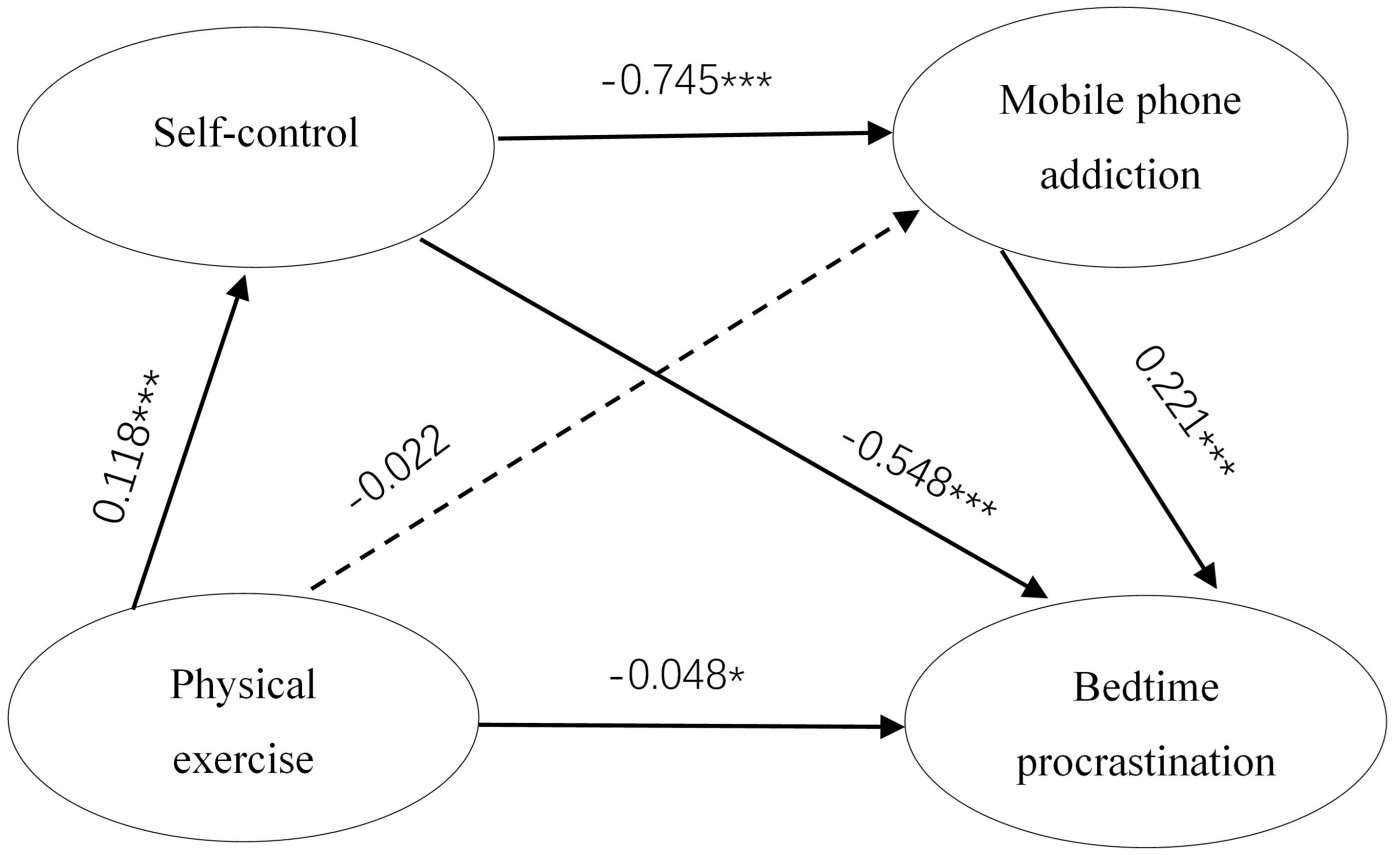
The chain mediating role of self-control and mobile phone addiction in the impact of physical exercise on bedtime procrastination. ***p < 0.001, **p < 0.01, *p < 0.05.

Multiple regression analysis revealed that physical exercise had a significant negative predictive effect on bedtime procrastination among college students (β = −0.048, 95% CI = [−0.095, −0.002]). Additionally, self-control exhibited a significant negative impact on mobile phone addiction (β = −0.745, 95% CI = [−0.807, −0.682]), and it also significantly predicted bedtime procrastination negatively (β = −0.548, 95% CI = [−0.642, −0.453]). Furthermore, mobile phone addiction was found to have a significant positive effect on bedtime procrastination among college students (β = 0.221, 95% CI = [0.144, 0.298]) (Figure 2; Table 2).

**Table 2:** The chain mediating model coefficients of self-control and mobile phone addiction in the impact of physical exercise on bedtime procrastination.

Predictive variables	Mobile phone addiction					Bedtime procrastination				
	β	*SE*	*t*	95%CI		β	*SE*	*t*	95%CI	
Grade	0.039	0.015	2.618**	0.010	0.068	0.053	0.018	2.995**	−0.018	−0.088
Physical exercise	−0.022	0.020	−1.130	−0.061	0.017	−0.048	0.024	−2.033*	−0.095	−0.002
Self-control	−0.745	0.032	−23.339***	−0.807	−0.682	−0.548	0.048	−11.366***	−0.642	−0.453
bedtime procrastination						0.221	0.039	5.616***	0.144	0.298
R^2^	0.386					0.314				
F	195.089***					106.444***				

***p < 0.001, **p < 0.01, *p < 0.05.

### 4.4 The mediating role of physical exercise

The deviation-corrected non-parametric percentile Bootstrap method was employed to examine the mediating effects. The results indicated that the chain mediation model demonstrated a significant total effect (95% CI = [−0.191, −0.083]) and a significant direct effect (95% CI = [−0.095, −0.002]), with effect sizes of −0.137 and −0.048, respectively. The mediating path of physical exercise → self-control → bedtime procrastination was significant (95% CI = [−0.095, −0.038]), with an effect size of −0.064, accounting for 47% of the total effect. This suggests that physical exercise can negatively influence college students’ bedtime procrastination through the mediating role of self-control. However, the mediating path of physical exercise → mobile phone addiction → bedtime procrastination was not significant, indicating that mobile phone addiction alone did not significantly mediate the relationship between physical exercise and bedtime procrastination. Notably, the chain mediation path of physical exercise → self-control → mobile phone addiction → bedtime procrastination was significant (95% CI = [−0.032, −0.010]), with an effect size of −0.019, accounting for 14% of the total effect (see Table 3). These findings suggest that physical exercise may reduce mobile phone addiction by enhancing self-control, thereby ultimately decreasing bedtime procrastination among college students.

**Table 3:** Chain mediation analysis of the effects of self-control and mobile phone addiction.

Pathway	Effect	Boot SE	95%CI Boot LLCI	95%CI Boot ULCI	Relative effect proportion
Direct effects					
Physical exercise → Bedtime procrastination	−0.048	0.024	−0.095	−0.002	35%
Indirect effects					
Physical exercise → Self-control → Bedtime procrastination	−0.064	0.015	−0.095	−0.038	47%
Physical exercise →Mobile phone addiction → Bedtime procrastination	−0.005	0.005	−0.015	0.004	4%
Physical exercise → Self-control → Mobile phone addiction → Bedtime procrastination	−0.019	0.006	−0.032	−0.010	14%
Total effects					
Physical exercise → Bedtime procrastination	−0.137	0.028	−0.191	−0.083	100%

## 5 Discussion

This study explored the influence of physical exercise on college students’ bedtime procrastination and the chain mediating role of self-control and mobile phone addiction in this process, explained the possible mechanisms of physical exercise on bedtime procrastination, deepened the research on the improvement of psychological quality of physical exercise, and provided theoretical basis and practical guidance for the prevention and intervention of bedtime procrastination among college students.

### 5.1 Physical exercise and bedtime procrastination

The results of this study indicate that physical exercise significantly and negatively predicts bedtime procrastination among college students. In other words, higher levels of physical exercise are associated with lower levels of bedtime procrastination. This finding supports Hypothesis 1 and aligns with previous research, further highlighting the important role of physical exercise in promoting healthier sleep behaviors among college students.

Numerous studies have indicated that physical exercise can significantly alleviate bedtime procrastination by regulating biological rhythms, enhancing neurophysiological states, and improving cognitive resources and emotional regulation abilities (Gong et al., 2024; Mu et al., 2024; Shen et al., 2023; Zhang et al., 2025). Regular physical exercise, on one hand, aids in synchronizing circadian rhythms, improving sleep quality (Shen et al., 2023), and subsequently reducing bedtime procrastination. Furthermore, physical exercise helps to relieve stress and anxiety accumulated during the day, mitigating emotional disturbances at night and enhancing the motivation to fall asleep (Sirois and Pychyl, 2013). On the other hand, as a highly disciplined activity, physical exercise strengthens time management and behavioral control abilities, thereby reducing procrastination (Contreras-Osorio et al., 2022). The findings of this study further confirm the beneficial role of physical exercise in enhancing sleep behavior, suggesting that its behavioral regulatory function should be emphasized in mental health interventions for college students.

### 5.2 Mediating role of self-control in the relationship between physical exercise and bedtime procrastination

In this study, the results indicate that self-control acts as a mediator in the relationship between physical exercise and bedtime procrastination. Specifically, engaging in physical exercise may enhance self-control among college students, which in turn reduces bedtime procrastination. The findings are consistent with prior researches. Several studies have established a significant positive correlation between physical exercise and self-control, suggesting that college students who participate in higher levels of physical exercise tend to exhibit stronger self-control abilities (Xu and Tang, 2024). Furthermore, research has shown a significant negative correlation between self-control and bedtime procrastination among college students, indicating that individuals with higher self-control are more likely to adhere to regular sleep schedules and demonstrate less procrastination at bedtime (Kamphorst et al., 2018; Kroese et al., 2014; You et al., 2020).

According to cognitive behavioral theory, physical exercise may indirectly inhibit bedtime procrastination by enhancing recovery from ego depletion. Research indicates that children who engage in regular exercise can improve their executive function, particularly inhibitory control (Best, 2010). This enhancement increases their likelihood of resisting the allure of bedtime entertainment, such as short videos and social media, thereby decreasing the incidence of bedtime procrastination. This finding suggests that physical exercise enhances cognitive resources such as self-control and executive function (Best, 2010; Mandolesi et al., 2018). Therefore, there exists a significant correlation between college students’ participation in physical exercise and the enhancement of their self-control abilities. Furthermore, physical exercise may indirectly influence bedtime procrastination by improving self-control. These findings clearly illustrate the mediating role of self-control in the relationship between physical exercise and bedtime procrastination among college students.

### 5.3 Mediating role of mobile phone addiction in the relationship between physical exercise and bedtime procrastination

This study found a correlation between physical exercise and mobile phone addiction, as well as between mobile phone addiction and bedtime procrastination. However, mobile phone addiction did not serve as a significant mediator in the impact of physical exercise on bedtime procrastination. These findings differ somewhat from the conclusions of certain previous studies, highlighting the need for a deeper understanding of the mechanisms underlying mobile phone addiction and bedtime procrastination.

According to the self-control resource model and cognitive behavioral theory, mobile phone addiction significantly impacts bedtime procrastination among college students. Research indicates that heightened academic pressure often leads students to compensate for psychological stress by using their mobile phones at night. This behavior disrupts their biological rhythms, delays sleep onset, and increases the likelihood of bedtime procrastination (Exelmans and Van den Bulck, 2016; Meng et al., 2024). Furthermore, numerous studies have demonstrated that physical exercise can indirectly enhance sleep quality by mitigating mobile phone addiction in college students (Guo et al., 2022; Shi et al., 2021). However, the current research findings indicate that mobile phone addiction does not significantly mediate the relationship between physical exercise and bedtime procrastination. This may be attributed to several factors: (1) The development of mobile phone addiction is relatively complex and is influenced by various psychological factors, such as anxiety, loneliness, and low self-control (Li et al., 2020). Merely increasing physical exercise may not be sufficient to mitigate mobile phone addiction, as existing research suggests that the beneficial effects of exercise may operate indirectly by enhancing individuals’ psychological traits, such as self-control or resilience, rather than exerting a direct influence on addictive behaviors themselves (Pirwani and Szabo, 2024). (2) The positive effects of physical exercise on sleep are more likely to be mediated through direct physiological mechanisms (e.g., regulation of melatonin secretion, stress reduction), rather than primarily through reductions in mobile phone addiction (Shah et al., 2023). Consequently, physical exercise may not necessarily impact bedtime procrastination through alterations in mobile phone usage behavior.

### 5.4 A chain mediating role of self-control and mobile phone addiction in the relationship between physical exercise and bedtime procrastination

Self-control and mobile phone addiction play a mediating role in the relationship between physical exercise and bedtime procrastination in the present study. Although direct evidence linking physical exercise, self-control, mobile phone addiction, and bedtime procrastination is lacking, existing studies have established a negative predictive relationship between self-control and mobile phone addiction. Specifically, individuals with lower self-control may struggle to suppress the impulse to use mobile phones, thereby increasing their risk of addiction (Cheng et al., 2023). This suggests that diminished self-control correlates with a higher likelihood of mobile phone addiction (Liu and Chen, 2023), which aligns with the self-control resources theory. Furthermore, research has demonstrated that enhancing willpower can mitigate bedtime procrastination by reducing mobile phone addiction (Bernecker and Job, 2020; Meng et al., 2024). Consequently, this study posits that college students can enhance their self-control through physical exercise, leading to a reduction in mobile phone addiction and a subsequent alleviation of bedtime procrastination.

This study found that in the model of physical exercise → mobile phone addiction → bedtime procrastination, physical exercise was negatively correlated with mobile phone addiction, indicating that physical exercise may influence mobile phone usage behavior through various mechanisms. When included in the chain mediation model of physical exercise → self-control → mobile phone addiction → bedtime procrastination, the mediating effect of mobile phone addiction was not statistically significant. A possible explanation for this is that self-control may dominate the pathway, potentially masking the indirect effects of phone addiction. According to self-determination theory (Deci, 2004), physical exercise may inhibit bedtime procrastination behavior by fulfilling individuals’ basic psychological needs, thereby enhancing self-control. In the chain mediation model of this study, self-control exhibited a significant mediating effect on bedtime procrastination, while the mediating effect of mobile phone addiction was not significant. This discrepancy suggests that physical exercise directly intervenes in procrastination behavior by improving individuals’ cognitive regulatory resources, rather than relying on the indirect regulation of mobile phone addiction.

Furthermore, self-control may exert a significant inhibitory effect on mobile phone addiction, thereby diminishing the independent pathway through which physical exercise influences bedtime procrastination via mobile phone addiction. These findings indicate that the direct effects of cognitive resources may surpass the indirect effects of behavioral dependencies, such as mobile phone addiction, within the framework of multiple mediations. Chain mediation model analysis reveals that when self-control and mobile phone addiction function as simultaneous mediators, overlapping effects may arise between them. The Bootstrap test further corroborated that the mediating effect size of self-control was significantly greater than that of the mobile phone addiction pathway, suggesting that physical exercise plays a more critical role in mitigating bedtime procrastination by enhancing the cognitive regulation benefits associated with self-control. This finding aligns with the social cognitive theory, which posits that cognitive factors predominantly influence behavior change (Bandura, 1986). It underscores the significance of individual self-control in behavioral interventions. While the mediating effect of mobile phone addiction is substantial in the model of physical exercise → mobile phone addiction → bedtime procrastination, the mediating effect value of this chain model diminishes due to competitive effects and the cumulative error from multiple pathways, resulting in no statistical significance. Given the various mediating mechanisms, future research could employ experimental designs, ecological momentary assessments, or longitudinal tracking methods to more precisely explore the dynamic relationships among physical exercise, self-control, and mobile phone addiction. This would enhance our understanding of the behavioral mechanisms underlying bedtime procrastination and inform the development and application of intervention strategies. In conclusion, this study posits that the impact of physical exercise on mobile phone addiction may be more indirect, as physical exercise potentially influences mobile phone addiction by affecting college students’ self-control.

This study suggests that interventions utilizing physical exercise to reduce bedtime procrastination among college students should prioritize the enhancement of self-control, a core element of psychological capital, rather than solely focusing on restricting mobile phone use. This approach aligns with the positive psychology principle of prioritizing empowerment rather than restriction (Seligman and Csikszentmihalyi, 2000), suggesting that the promotion of healthy behaviors should emphasize the development of internal psychological resources rather than the mere regulation of superficial behaviors. Consequently, colleges and universities should actively encourage extensive participation in physical exercise among students and implement systematic strategies to enhance their self-control abilities. This, in turn, will help mitigate mobile phone addiction and more effectively address the issue of bedtime procrastination through the positive effects of physical exercise.

## 6 Conclusion

Physical exercise exerts a significant influence on bedtime procrastination among college students, with self-control and mobile phone addiction functioning as chain mediators in this relationship.

## 7 Limitations and perspectives

First, the cross-sectional self-reporting questionnaire utilized in this study limited the determination of causality. Although some confounding variables were controlled for in the statistical model, the influence of other potential confounding factors could not be entirely excluded. Future studies should employ longitudinal tracking designs and experimental intervention methods to test the causal effect of physical exercise on bedtime procrastination, further clarifying the time series relationship between self-control and mobile phone addiction, and exploring the dynamic mechanisms of change between these variables. Second, the study sample comprised only 1,000 college students from Shanghai, Tianjin, Zhejiang, and Anhui provinces in China, which may limit the representativeness of the sample and affect the external validity of the conclusions. Future research should expand the sample coverage to include more university students from diverse regions to enhance the representativeness and generalizability of the findings. College students from varying cultural backgrounds, academic years, or fields of study may exhibit significant differences in their physical exercise habits, sleep behaviors, and mobile phone usage patterns, necessitating further comparison and verification in subsequent studies. Finally, it is recommended to consider more detailed behavioral measurement tools, such as wearable devices to record exercise and sleep behaviors, combined with digital behavior tracking technology to analyze mobile phone usage data. This approach would improve the objectivity and ecological validity of the data collected.

## Data Availability

The raw data supporting the conclusions of this article will be made available by the authors, without undue reservation.
